# Preliminary Education of Medical Students

**Published:** 1876-05

**Authors:** 


### Editorial.

### Preliminary Education of Medical Students.

We clip the following from the Annual Announcement of the Buffalo Medical College for the session of 1876–'77:

" Upon Marticulating, students will be required to pass an examination which shall exhibit evidence of possessing that preliminary education necessary to the intelligent discharge of a Physician's duties.

" Certificates of attendance at, or graduation from Literary Colleges, Scientific Schools or Medical Colleges, or diplomas from High Schools or Academies, will exempt from this examination."

This is a step in advance which we are glad to welcome. While it will not, to some, seem a sufficiently high standard, they must remember that few reforms are accomplished at once, there are the important initiatory steps which serve as much to bring about the desired end as the grand final move. Medical students have heretofore been exempt from all inquiries as to their fitness to study medicine, and to require of them at once to pass an examination as difficult as some propose, is simply absurd, especially so when the whole profession are not united on the subject.

The examination of students entering upon the study of medicine should properly belong to the colleges. Where this is not enforced, the next best move in advance is to have it undertaken by the County Societies. The Medical Society of the State of New York has proposed, and a few County Societies under its recommendation have adopted, resolutions forbidding any member of those organizations receiving students who have not passed an examination before a board appointed by those societies. As a step forward in medical reform this is an important move; it is, however, in many respects defective. The Medical Society of the County of Erie, for instance, has a Primary Board for the examination of medical students, the societies of several of the counties in close proximity have none, it is then, perfectly evident that students from the counties where no examination is required can demand entrance to medical colleges on the same conditions as those who have a certificate of proper qualification. But, should all the societies of the State require students to appear before their Boards of Examiners, it is evident that the standard of excellence, and the strictness of the examination would vary with the different boards, and some students would be admitted to study in evident injustice to others who had been debarred from that right.

The Primary Board of the Medical Society of the County of Erie, in their annual report presented January 11th, 1876, use the following language: *

"We must bear in mind too, that the prime object of medical schools is not to benefit the profession at large, and to promote medical science, but the pecuniary and professional advantage of their faculties. And we have no right to expect them to take an active interest in a work of reform, which if thoroughly carried out, might result to their disadvantage."

If the gentlemen who wrote that report are in earnest, we are sorry they have no higher appreciation of their professional brethren who are engaged in medical teaching. It is true there are schools of medicine, hospitals and various medical charities, which are organized solely for the purpose of advertising the medical men connected with them, and the fact is certainly to be lamented that the ardent rivalry of some, or the desire of others for advertisement has produced so many medical colleges; colleges, like a Western institution now before the profession in no enviable light, which receive students from almost any source for almost nothing, and graduate them in almost no time.

We make the assertion, however, that the majority of medical teachers, those who to-day are held in respect by the mass of the profession, will gladly welcome any move which will elevate the mental attainments of those whom they receive as students. The time is not far distant, we hope, and already some of the medical schools are making preparations for the change, when the schools will take the matter entirely out of the hands of the profession. When private preceptors will be a thing of the past. Then the student will have to pass an entrance examination to a medical college as he would to a literary college, be required to spend a definite period in the pursuit of a graded course of study under a competent board of instructors, and pass an examination which shall be alike for all colleges, and be conducted by a State Board of Examiners. In accomplishing these reforms the colleges need the support of the general profession, they appreciate the difficulties of the situation better than any class of outside "reformers" can; whose motto is most excellent, but whose motives will not merit similar praise. B.

:o:

### Sanitary Condition of Philadelphia.

We have received the following circular to which we direct the attention of our readers:

UNITED STATES CENTENNIAL COMMISSION. BUREAU OF MEDICAL SERVICE.

Owing to the very large number of persons who contemplate a visit to Philadelphia during the coming summer, it seems important that the utmost publicity should be given to all facts bearing on the sanitary condition of the city.

* Transactions of the Medical Society of the County of Erie, Buffalo, 1876. Pamphlet.

